# Ononin Sensitizes Papillary Thyroid Carcinoma Cells to Cisplatin by Repressing DNA Damage Response via E2F2

**DOI:** 10.1155/ije/8372639

**Published:** 2026-02-14

**Authors:** Xian Deng, Xin Qian, Lian Cheng, Dehui Qiao, Rongjia Zhang, Xu Li

**Affiliations:** ^1^ Department of General Surgery (Thyroid Surgery), The Affiliated Hospital, Southwest Medical University, Luzhou, China, swmu.edu.cn; ^2^ Nuclear Medicine and Molecular Imaging Key Laboratory of Sichuan Province, Luzhou, China; ^3^ Cardiovascular and Metabolic Diseases Key Laboratory of Luzhou, Luzhou, China

**Keywords:** cisplatin, DNA damage response, E2F2, ononin, papillary thyroid carcinoma

## Abstract

Ononin, a natural isoflavone glycoside, has demonstrated antitumor activity in several malignancies, but its role in papillary thyroid carcinoma (PTC) remains unclear. Here, we show that ononin sensitizes PTC cells to cisplatin by attenuating the DNA damage response (DDR) through the E2F2/MDC1 axis. Ononin treatment suppresses cell proliferation, colony formation, and epithelial–mesenchymal transition in PTC cell lines. Pretreatment with ononin significantly reduces the half‐maximal inhibitory concentration (IC_50_) of cisplatin, indicating its potential as a chemosensitizing agent. Mechanistically, cisplatin resistance in PTC cells is associated with enhanced DDR and homologous recombination (HR) repair driven by E2F2. Ononin downregulates E2F2 expression, leading to reduced expression of MDC1, a key mediator of DDR and HR. Functional assays confirm that ononin‐induced repression of E2F2 impairs DNA repair capacity and increases cisplatin sensitivity in cisplatin‐resistant PTC cells. These findings identify ononin as a promising adjuvant candidate for overcoming cisplatin resistance in PTC by targeting the E2F2/MDC1‐dependent DDR pathway.

## 1. Introduction

Thyroid carcinoma is one of the most common malignant tumors of the endocrine system. More than 90% of endocrine malignancies are thyroid carcinoma [1]. Over the last few decades, the incidence of thyroid carcinoma has been increasing rapidly worldwide. Based on clinical and pathological features, thyroid carcinoma is categorized into papillary thyroid carcinoma (PTC), follicular thyroid carcinoma, and anaplastic thyroid carcinoma (ATC) [2]. PTC accounts for approximately 80%–85% of all thyroid carcinoma cases [3]. Although most patients with PTC have a favorable prognosis and long‐term survival owing to its typically indolent clinical behavior, a subset of PTC patients display highly aggressive clinical features, which shorten long‐term survival and worsen prognosis [4]. The main PTC treatment is surgery with complete removal of the thyroid gland. However, in cases of recurrence and in patients with resistance to radioactive iodine therapy, systemic chemotherapy is considered [5]. Resistance to current chemotherapeutic regimens in PTC has been reported; therefore, our study aims to elucidate the underlying molecular mechanisms and to provide potential strategies to overcome this resistance.

Ononin (C_22_H_22_O_9_) is an isoflavone glycoside compound derived from ononis, soybean, *Glycyrrhiza uralensis*, red clover fruits, and other herbal plants [6]. Recent studies have shown that ononin has multiple bioactivities, including antiproliferation, anti‐inflammation, and antimetastasis, and thus, ononin is considered a potential chemopreventive agent [7]. Ononin displays tumor‐suppressive effects by inhibiting the Ras/Raf/Erk pathway [8], NF‐κB pathway [9], and PI3K/AKT/mTOR pathway [10] in different cancers. However, the effects of ononin in thyroid carcinoma, especially in PTC, remain largely unknown. In this study, we show that ononin inhibits the proliferation of PTC cells and sensitizes PTC cells to cisplatin treatment by modulating DNA damage response (DDR) and homologous recombination (HR) repair via the E2F2/MDC1 axis.

Human cancer cells can become tolerant to prolonged exposure to chemotherapeutic drugs [11]. The acquired resistance is mainly caused by activation of DDR pathways. In normal cells, DDR is essential for the maintenance of genome stability and integrity, but in cancer cells, DDR can represent an obstacle to chemotherapy, as DDR attenuates the DNA damage effect of chemotherapy drugs. Several inhibitors targeting ATM, PARP1, and ATR are currently in clinical trials [12–14]. Therefore, repression of DDR is one of the strategies to overcome chemotherapy resistance. Here, we show that ononin sensitizes PTC cells to cisplatin by modulating DDR.

## 2. Materials and Methods

### 2.1. Cell Lines, Plasmids, and Reagents

In this study, we purchased human PTC cell lines TPC‐1 and B‐CPAP from Millipore (USA). The cells were cultivated in RPMI‐1640 medium with 10% fetal bovine serum (FBS), 100 U/mL penicillin, and 100 µg/mL streptomycin, at 37°C in a humidified atmosphere of 5% CO_2_. To generate cisplatin‐resistant cells (TPC‐1^CR^ and B‐CPAP^CR^), TPC‐1 and B‐CPAP cells were initially seeded into a 10‐cm dish (5 × 10^6^ cells/dish) with full RPMI‐1640 medium. Cells were then continuously treated with 1.0, 2.0, 4.0, 8.0, 16.0, and 32.0 µmol/L cisplatin (dissolved in DMSO) every 2‐3 days. Harvest cells survived from 32.0 µmol/L cisplatin treatment as resistant cells. To achieve E2F2 and MDC1 overexpression, we cloned protein‐coding region of E2F2 and MDC1 into pcDNA3.1 (+) vector and the inserts were validated by Sanger sequencing. The constructed vectors were named as pcDNA‐E2F2 and pcDNA‐MDC1, respectively. For transfection, cells were seeded into 6‐well plates (2 × 10^5^ cells/well) one day before transfection. The transfection procedure was strictly followed by the protocol of Lipofectamine 2000 (Invitrogen, USA). The final concentration of plasmid was 1 µg/mL. After 48‐h transfection, cells were subjected to the following analysis. Ononin (formononetin 7‐*O*‐β‐*D*‐glucopyranoside, C_22_H_22_O_9_) was purchased from Merck (75375, USA) and was dissolved in DMSO at 100 µmol/L as a stock solution. Anti‐E2F2, anti‐MDC1, and anti‐β‐actin primary antibodies were obtained from ProteinTech.

### 2.2. RNA Extraction and Real‐Time Quantitative PCR (Q‐PCR)

The total RNA was isolated from cells by using the High Pure RNA Isolation Kit (Roche, Switzerland) according to the provided manufacturer’s manual. A total of 100‐ng RNAs were subjected to generate a cDNA library by using Verso cDNA Synthesis Kit (Thermo Fisher, USA). Q‐PCR was performed on Applied Biosystems (USA) with SYBR Green reagent. GAPDH served as an internal negative control. The changes in gene expression were calculated by using the comparative Ct method. The primers used in Q‐PCR are listed in Table 1.

**TABLE 1:** Primers used in qPCR analysis.

Target	Forward 5′‐3′	Reverse 5′‐3′
E2F2	CTC​TCT​GAG​CTT​CAA​GCA​CCT​G	CTT​GAC​GGC​AAT​CAC​TGT​CTG​C
MDC1	GCA​AGA​TGC​CAC​CTG​CTG​AGA​A	GCT​TCA​GGT​ACT​GTA​GGA​GGC​A
CDH1	GCC​TCC​TGA​AAA​GAG​AGT​GGA​AG	TGG​CAG​TGT​CTC​TCC​AAA​TCC​G
VIM	AGG​CAA​AGC​AGG​AGT​CCA​CTG​A	ATC​TGG​CGT​TCC​AGG​GAC​TCA​T
SNAI1	TGC​CCT​CAA​GAT​GCA​CAT​CCG​A	GGG​ACA​GGA​GAA​GGG​CTT​CTC
SNAI2	ATC​TGC​GGC​AAG​GCG​TTT​TCC​A	GAG​CCC​TCA​GAT​TTG​ACC​TGT​C

### 2.3. IC_50_ Determination, Cell Proliferation, and Colony Formation Assays

3‐(4,5‐Dimethylthiazol‐2‐yl)‐2,5‐diphenyltetrazolium bromide (MTT, M6494, Thermo Fisher) was used to determine IC_50_s of drugs and the proliferation rate of cells according to the protocol provided by Thermo Fisher (USA). To evaluate colony formation capability, cells were seeded into 6‐well plates at a density of 500 cells/well and cultivated for at least 2 weeks or until colonies could be observed with the eyes. Cells were fixed by 100% methanol for 20 min and then stained by 0.5% crystal violet for 30 min. The number of colonies was quantified by ImageJ software.

### 2.4. Chromatin Immunoprecipitation‐qPCR (qChIP) Analysis

qChIP analysis was performed by using iDeal ChIP‐qPCR Kit (C01010180, Diagenode, Belgium). DNA was isolated from 4 million cells and fragmented by sonication for 15 s for 25 rounds on ice in ethanol. After decrosslinking, chromatin was subjected to qPCR analysis. The primers used in the analysis are listed in Table 2.

**TABLE 2:** Primers used in qChIP analysis.

Target	Forward 5′‐3′	Reverse 5′‐3′
MDC1	AACCCACTACCGCTTGCC	CGAGGAAAGGCGCTCTG
16q22	CCT​TCA​TTG​GGA​TCA​CCA​CG	AGG​AGA​TGA​GTA​CCA​GCA​GGT​TG

### 2.5. Immunofluorescence (IF)

DNA damage was determined by IF. Cells were seeded on the cover slips in 12‐well plates with the indicated treatments. Cells were fixed by 4% PFA at room temperature for 20 min and then incubated with 0.1% Triton X‐100 for 5 min. 1% BSA in PBS was used for blocking for 30–60 min at room temperature. The cover slips were incubated with primary antibody (γH2AX) overnight at 4°C followed by Alexa Fluor 555 secondary antibody incubation for 1 h at room temperature. The nucleus was stained by DAPI for 10 min. The cover slips were observed using Zeiss LSM 800 (Germany).

### 2.6. HR and Nonhomologous End Joining (NHEJ) Repair Assay

Cells were transfected with DR‐GFP and pCBAScel vectors for HR repair determinations. Cells were transfected with EJ5‐GFP and pCBAScel vectors for NHEJ repair determinations. All vectors were obtained from Addgene (USA). After 48‐h transfection, cells were subjected to flow cytometry (FACS) analysis. The efficiency of HR or NHEJ was represented by the percentage of GFP‐positive cells after transfection.

### 2.7. Western Blots

The total protein was isolated from cells by using RIPA lysis buffer (Beyotime, China) following the indicated treatments in the study. For patient samples, cut 5‐ to 10‐µm sections from the paraffin‐embedded tissue blocks using a microtome. 1 mL of xylene was used for deparaffinization and the harvested pellet was centrifuged for 5 min and subsequently washed pellet with 100% ethanol. Next, performed rehydration steps by washing with 95%, 70%, and 50% ethanol, followed by distilled water. RIPA lysis buffer (Beyotime, China) was used for protein isolation. For the cell, the lysate was sonicated for 7 min and harvested the supernatant (protein part) after centrifuging for 15 min at 4°C. Protein was quantified by using the BCA protein assay, and a total of 30 µg of protein was loaded and separated by 8.5% sodium dodecyl sulfate (SDS) polyacrylamide gel electrophoresis. Protein was then transferred onto PVDF membranes (Millipore, USA) and followed by incubation with primary antibody (overnight at 4°C) and secondary antibody (1 h at room temperature). The ECL (Millipore) system was used and imaged through the LI‐COR Odyssey FC imaging system. The information of antibodies used was listed below: anti‐MDC1 (1:1000, 24721‐1‐AP, Proteintech), anti‐E2F2 (1:1000, ab318956, Abcam), and anti‐β‐actin (1:1000, SC‐47778, Santa Cruz).

### 2.8. Patient Samples

Primary tumor specimens were obtained from 12 patients with histologically confirmed PTC who received cisplatin‐based chemotherapy at the Affiliated Hospital of Southwest Medical University over a 3‐year period (from 03.2020 to 03.2023). The study was conducted in accordance with the Declaration of Helsinki and was approved by the Institutional Ethics Committee of Southwest Medical University (approval no. SMU25125). Written informed consent was obtained from all participants prior to sample collection.

### 2.9. Bioinformatics Analysis

The patient cohort GSE50901 was downloaded from the GEO database (https://www.ncbi.nlm.nih.gov/geo/), and the Limma package (v3.40.2) in R was used to screen the differentially expressed genes (DEGs). The false‐positive candidates were excluded by applying adjusted *p* values. The criteria of screening DEGs were defined as “Adjusted *p* < 0.05 and log2 (fold change) > 1 or log2 (fold change) < −1.” To analyze potential functions of DEGs, functional enrichment analysis was performed, including gene ontology (GO), molecular function (MF), biological process (BP), and cellular component (CC). The ClusterProfiler package in R was used to enrich the KEGG pathways.

### 2.10. Statistics

Statistical analyses were performed using GraphPad Prism (Version 9.0; GraphPad Software). Unless otherwise specified, data are presented as mean ± standard deviation (SD) from three independent in vitro experiments. Comparisons between two groups were assessed using an unpaired two‐tailed Student’s *t*‐test. Comparisons among multiple groups were evaluated using two‐way analysis of variance (ANOVA), followed by appropriate post hoc tests when indicated. A *p* value < 0.05 was considered statistically significant.

## 3. Results

### 3.1. Ononin Sensitizes PTC Cells to Cisplatin Treatment

**FIGURE 1:**
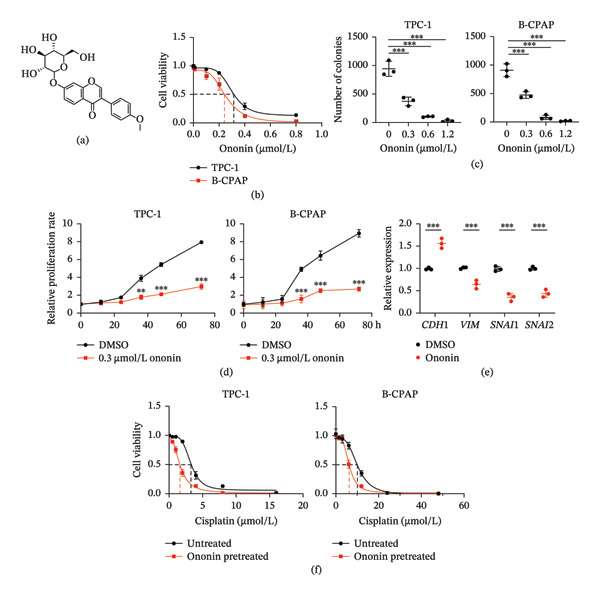
Ononin sensitizes PTC cell lines to cisplatin treatment. (a) The structure of ononin. (b) The IC_50_ of ononin in TPC‐1 and B‐CPAP cells tested by MTT assay. (c) The colony formation capability of TPC‐1 (left) and B‐CPAP (right) cells following treatments of ononin with different concentrations for 48 h. (d) The cell proliferation rate of TPC‐1 (left) and B‐CPAP (right) cells following 0.30 µmol/L ononin treatment for 24 h, determined by MTT assay. (e) The expression of EMT genes was determined by qPCR analysis in TPC‐1 and B‐CPAP cells following 0.30 µmol/L ononin treatment for 24 h. (f) The IC_50_ of cisplatin was determined in TPC‐1 (left) and B‐CPAP (right) cells with or without ononin pretreatment (24 h) by MTT assay. In panels b–f, the mean + SD (*n* = 3) is provided with ^∗∗^ *p* < 0.01 and ^∗∗∗^ *p* < 0.001.

To investigate the effect of ononin (Figure 1(a)) in PTC, we first determined the half‐maximal inhibitory concentration (IC_50_) of ononin in TPC‐1 and B‐CPAP cells. The IC_50_ of ononin was 0.30 ± 0.09 µmol/L in TPC‐1 cells and 0.25 ± 0.13 µmol/L in B‐CPAP cells (Figure 1(b)). The concentration 0.30 µmol/L was used in subsequent experiments for both TPC‐1 and B‐CPAP cells. In addition, Ononin suppressed the colony formation capacity of TPC‐1 and B‐CPAP cells in a concentration‐dependent manner (Figure 1(c)). The proliferation rate of TPC‐1 and B‐CPAP cells was also significantly reduced by ononin (Figure 1(d)). Interestingly, ononin exerted suppressive effects on epithelial–mesenchymal transition (EMT), as evidenced by increased expression of *CHD1* and decreased expression of mesenchymal marker genes, such as *VIM*, *SNAI1*, and *SNAI2* in TPC‐1 cells (Figure 1(e)). Next, we examined whether ononin treatment affects the response to cisplatin, which is commonly used in the treatment of PTC patients. Pretreatment of ononin for 24 h sensitized TPC‐1 and B‐CPAP cells to cisplatin (Figure 1(f)). Therefore, ononin is a promising agent that suppresses PTC progression and may serve as an adjuvant compound to enhance cisplatin sensitivity in PTC.

### 3.2. Generation of Cisplatin‐Resistant PTC Cells

**FIGURE 2:**
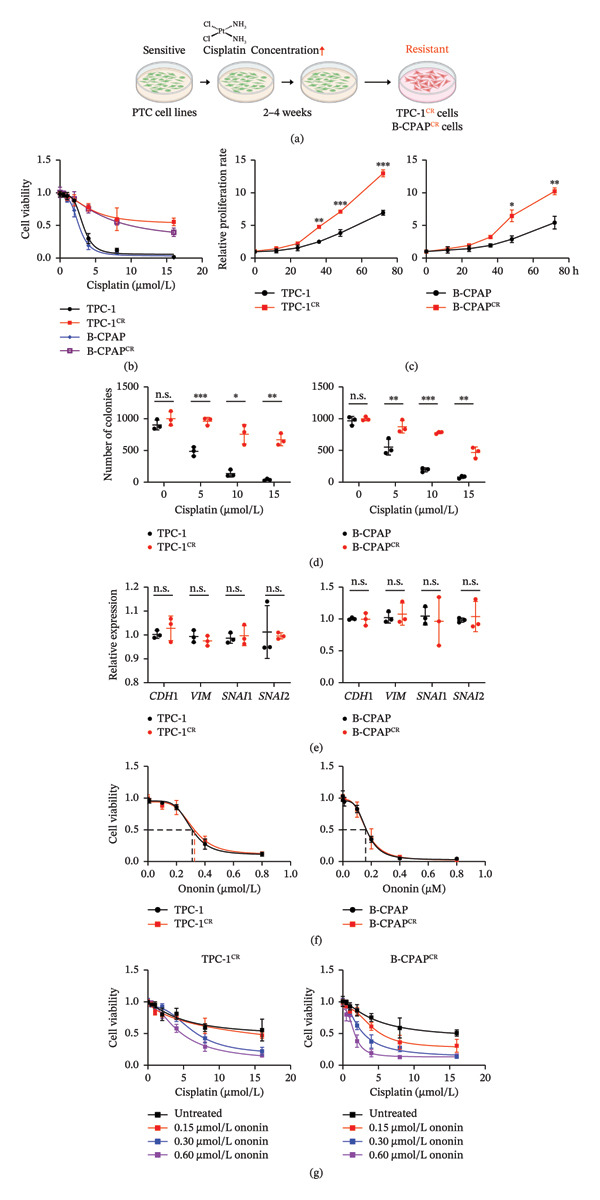
Generation of cisplatin‐resistant PTC cell lines. (a) The procedure for generating cisplatin‐resistant cells. Green: TPC‐1 or B‐CPAP cells sensitive to cisplatin; red: TPC‐1 or B‐CPAP cells resistant to cisplatin. (b) Cell viability of TPC‐1^CR^ and B‐CPAP^CR^ cells treated with cisplatin was validated by MTT assay. (c) The cell proliferation rate of parental and cisplatin‐resistant cells was determined by MTT assay, left panel: TPC‐1 and TPC‐1^CR^, right panel: B‐CPAP and B‐CPAP^CR^. (d) The colony formation capability of TPC‐1/TPC‐1^CR^ (left) and B‐CPAP/B‐CPAP^CR^ (right) cells following cisplatin treatment for 12 h. (e) The expression of EMT genes was determined by qPCR in TPC‐1/TPC‐1^CR^ (left) and B‐CPAP/B‐CPAP^CR^ (right) cells. (f) The IC_50_ of ononin in TPC‐1/TPC‐1^CR^ (left) and B‐CPAP/B‐CPAP^CR^ (right) cells tested by MTT assay. (g) The IC_50_ of cisplatin was determined in TPC‐1^CR^ (left) and B‐CPAP^CR^ (right) cells treated with ononin at different concentrations for 24 h. In panels b–g, the mean ± SD (*n* = 3) is provided with ^∗^ *p* < 0.05, ^∗∗^ *p* < 0.01, and ^∗∗∗^ *p* < 0.001.

To further validate the effect of ononin as a cisplatin sensitizer, we generated cisplatin‐resistant TPC‐1 and B‐CPAP cells (TPC‐1^CR^ and B‐CPAP^CR^, respectively) by continuous treatment with cisplatin for 2–4 weeks. The procedure is summarized in Figure 2(a). The IC_50_ values of TPC‐1^CR^ and B‐CPAP^CR^ cells were significantly increased compared with those of their parental cells (Figure 2(b)). In addition, TPC‐1^CR^ and B‐CPAP^CR^ cells displayed enhanced cell proliferation capability (Figure 2(c)) and colony formation capacity (Figure 2(d)) compared with parental cells, whereas the expression of EMT maker genes remained unchanged (Figure 2(e)). Notably, both TPC‐1^CR^ and B‐CPAP^CR^ cells remained sensitive to ononin treatment (Figure 2(f)). Upon ononin treatment, TPC‐1^CR^ and B‐CPAP^CR^ cells became re‐sensitized to cisplatin and this effect was dependent on the concentration of ononin (Figure 2(g)).

### 3.3. p53‐Associated Genes Are Involved in Cisplatin Resistance

To explore how ononin suppresses cisplatin resistance in PTC cell lines, we analyzed a PTC patient cohort (GSE50901) to clarify deregulated genes and pathways in PTC. We first screened for DEGs in PTC (Figure 3(a)) and subjected these DEGs to KEGG enrichment analysis (Figure 3(b)) and gene set enrichment analysis (GSEA) (Figure 3(c)). BPs related to wound healing, extracellular structure organization, and extracellular matrix organization were enriched in KEGG analysis (Figure 3(b)), which may at least partially explain the reduced EMT observed upon ononin treatment in PTC cell lines. GSEA revealed that genes in p53 downstream pathways were upregulated in PTC (Figure 3(c)). Furthermore, the expression profile of these p53 downstream genes in GSE50901 was consistent with that obtained from TCGA–THCA (Figure 3(d)). Therefore, activated DDR pathways may contribute to cisplatin resistance in PTC cell lines by enhancing the repair of cisplatin‐induced DNA breaks.

FIGURE 3p53‐associated genes are involved in cisplatin resistance. (a) The differentially expressed genes (DEGs) were screened in GSE50901 patient cohorts. Red dots: upregulated genes > 2.0‐fold; Blue dots: downregulated genes < 0.5‐fold. The upregulated p53‐associated genes were indicated in the volcano plot. (b) KEGG analysis of DEGs screened from GSE50901. (c) GSEA analysis of DEGs obtained from GSE50901. (d) The expression of p53‐associated genes selected from Figure 3(a) was validated in TCGA–THCA cohort. ^∗∗∗^ *p* < 0.001.

**Figure:**
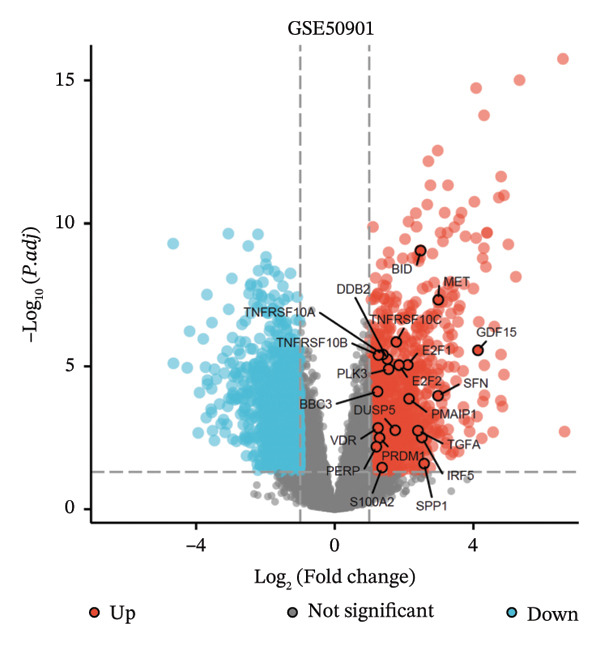
(a)

**Figure:**
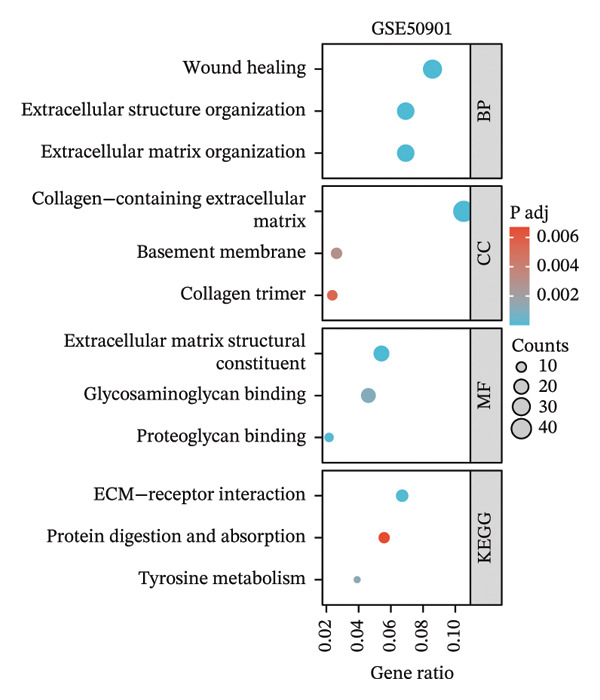
(b)

**Figure:**
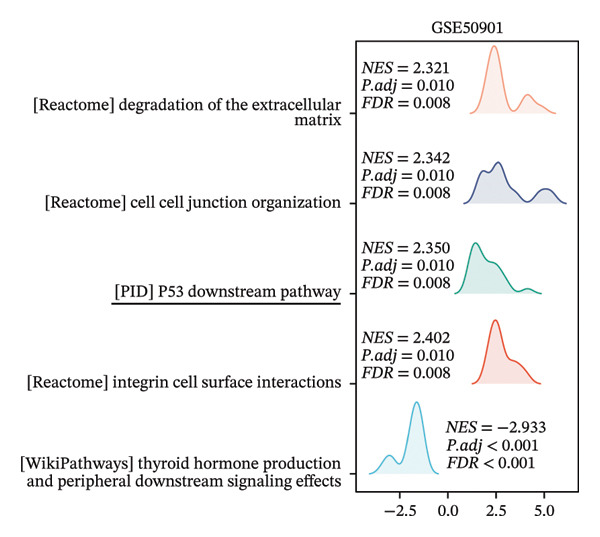
(c)

**Figure:**
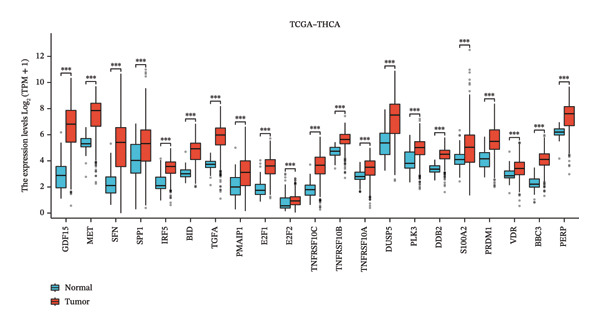
(d)

### 3.4. Ononin Sensitizes PTC Cell Lines to Cisplatin by Repressing E2F2

We next asked whether the genes selected from Figure 3(d) are regulated by ononin. Gene expression was examined in TPC‐1^CR^ and B‐CPAP^CR^ cells after 24 h of ononin treatment compared with untreated controls. Interestingly, only E2F2 expression was markedly reduced by ononin in cisplatin‐resistant cells (Figure 4(a)), indicating that ononin may sensitize cells to cisplatin by repressing E2F2. To validate that ononin overcomes cisplatin resistance via E2F2, we treated TPC‐1^CR^ and B‐CPAP^CR^ cells with ononin while ectopically expressing E2F2 for 48 h. E2F2 expression remained high following ectopic E2F2 vector transfection, even in the presence of ononin (Figure 4(b)). Cells overexpressing E2F2 remained resistant to cisplatin despite ononin pretreatment (Figure 4(c)). These results indicate that downregulation of E2F2 is required for ononin‐mediated sensitization of TPC‐1^CR^ and B‐CPAP^CR^ cells to cisplatin. In addition, ectopic E2F2 abrogated the inhibitory effect of ononin on cell proliferation (Figure 4(d)). Collectively, these findings demonstrate that ononin overcomes cisplatin resistance in PTC cell lines by repressing E2F2.

FIGURE 4Ononin sensitizes TPC‐1 cells to cisplatin by repressing E2F2. (a) E2F2 expression determined by qPCR (left) and western blot (right) analysis following 0.30 µmol/L ononin in TPC‐1^CR^ and B‐CPAP^CR^ cells. (b) The ectopic E2F2 validated by western blot after transfection of pcDNA‐E2F2 for 48 h. (c) The IC_50_ of cisplatin was determined in TPC‐1^CR^ (left) and B‐CPAP^CR^ (right) cells treated with 0.30 µmol/L ononin with or without ectopic E2F2. (d) The proliferation rate was determined in TPC‐1^CR^ (left) and B‐CPAP^CR^ (right) cells treated by 0.30 µmol/L ononin with or without ectopic E2F2. In panels A, c–d, the mean ± SD (*n* = 3) is provided with ^∗∗∗^ *p* < 0.001.

**Figure:**
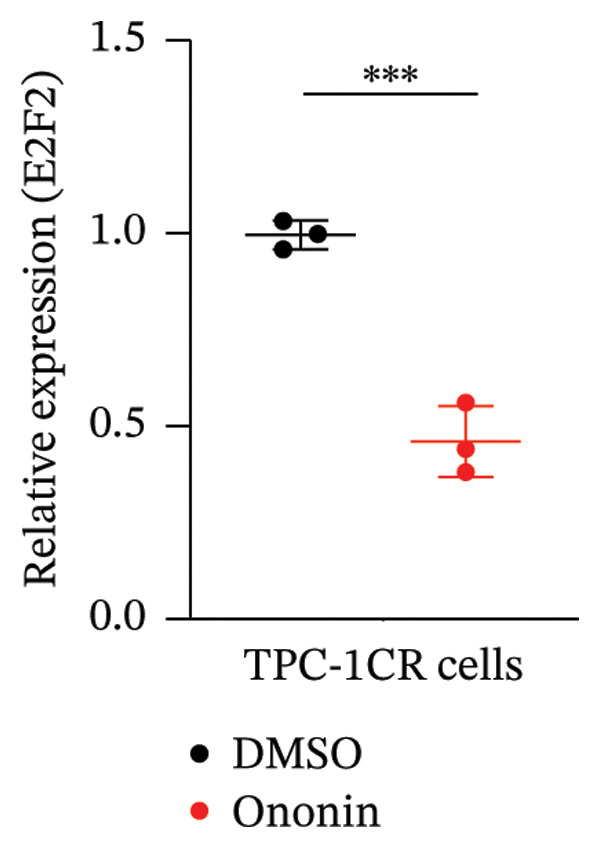
(a)

**Figure:**
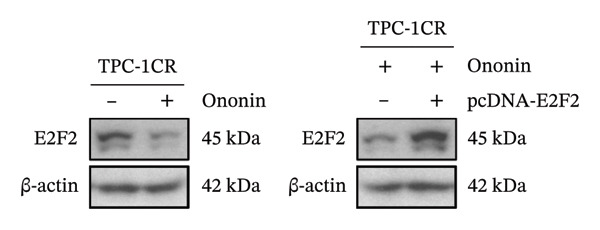
(b)

**Figure:**
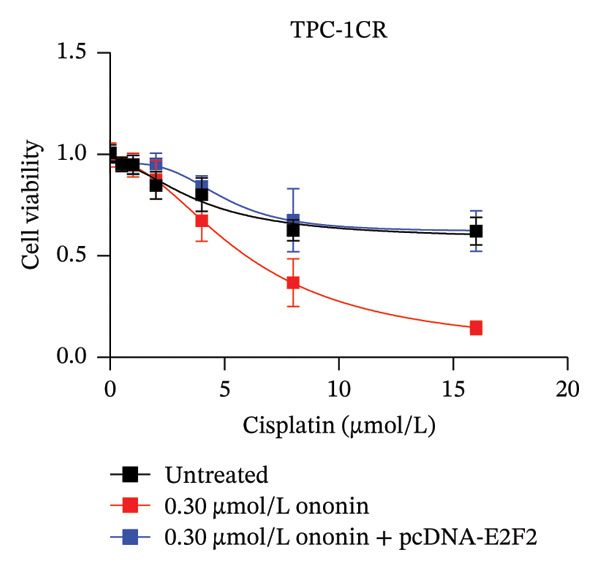
(c)

**Figure:**
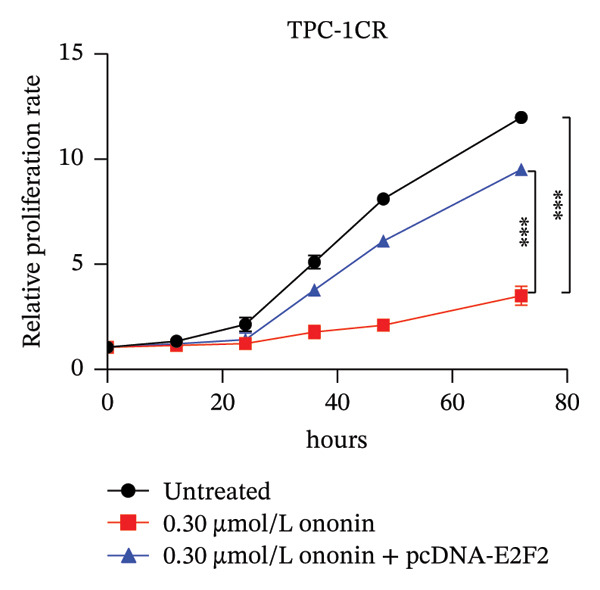
(d)

### 3.5. DNA Damage Response is Blocked by Ononin

**FIGURE 5:**
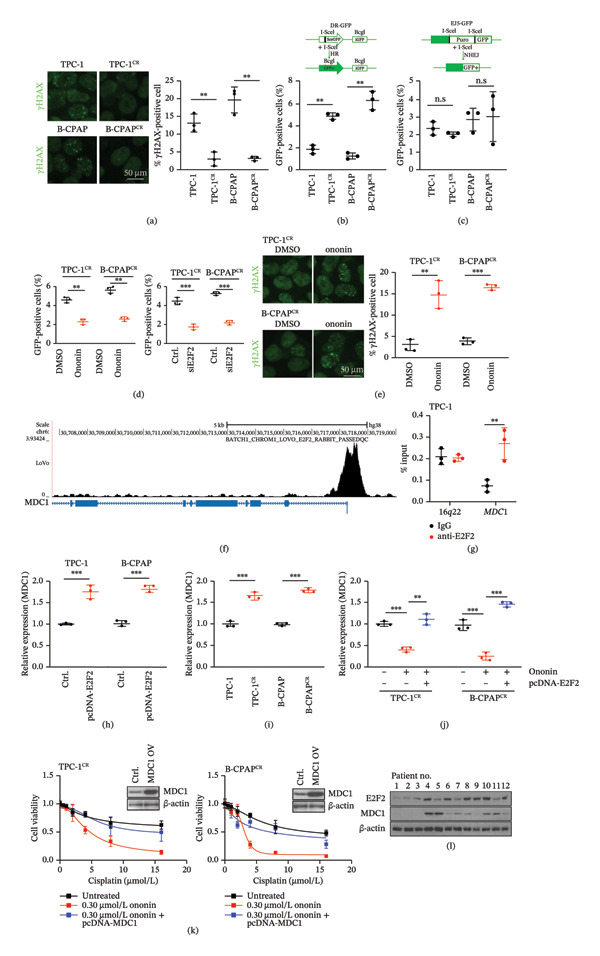
DNA damage response is blocked by ononin. (a) DNA damage determined by γH2AX staining in PTC parental and cisplatin‐resistant cells. The quantification was presented by the percentage of γH2AX‐positive cells. Scale bar: 50 µm. (b) HR repair efficiency was determined by FACS analysis for GFP‐positive cells (upper). HR repair efficiency evaluated in cisplatin‐sensitive and cisplatin‐resistant cells, respectively (lower). (c) NHEJ repair efficiency was determined by FACS analysis for GFP‐positive cells (upper). NHEJ repair efficiency evaluated in cisplatin‐sensitive and cisplatin‐resistant cells, respectively (lower). (d) HR repair efficiency determined in TPC‐1^CR^ and B‐CPAP^CR^ cells following 0.30 µmol/L ononin treatment for 24 h (left). HR repair efficiency determined in TPC‐1^CR^ and B‐CPAP^CR^ cells following E2F2 silencing for 48 h (right). (e) DNA damage determined by γH2AX staining in TPC‐1^CR^ and B‐CPAP^CR^ cells following 0.30 µmol/L ononin treatment for 24 h. The quantification was presented by the percentage of γH2AX‐positive cells. Scale bar: 50 µm. (f) E2F2 ChIP‐Seq analysis for MDC1. E2F2 peaks appeared at the promoter of MDC1 gene. (g) The target between E2F2 and MDC1 was validated by qChIP analysis. 16q22 served as a negative control. (h) MDC1 mRNA expression following ectopic E2F2 expression in TPC‐1 and B‐CPAP cells. (i) The basal level of MDC1 mRNA expression in cisplatin‐sensitive and cisplatin‐resistant cells. (j) The expression of MDC1 mRNA in TPC‐1^CR^ and B‐CPAP^CR^ cells following 0.3 µmol/L ononin treatment for 24 h with or without ectopic E2F2. (k) The IC_50_ of cisplatin was determined in TPC‐1^CR^ (left) and B‐CPAP^CR^ (right) cells treated by 0.30 µmol/L ononin with or without ectopic MDC1. The ectopic MDC1 was validated by western blot analysis. (l) MDC1 and E2F2 protein levels of PTC patients (*n* = 12) were determined by western blot. Patient Nos. 1–3 are cisplatin‐sensitive, and patient Nos. 4–12 are cisplatin‐resistant. In panels a–e, g–k, the mean ± SD (*n* = 3) is provided with ^∗∗^ *p* < 0.01, ^∗∗∗^ *p* < 0.001, and n.s: not significant.

Since E2F2 is involved in the p53‐regulated pathway and DDR [15], we next examined whether ononin regulates cisplatin resistance by modulating DDR via E2F2. Both TPC‐1^CR^ and B‐CPAP^CR^ cells displayed a lower level of DNA damage, as indicated by fewer γH2AX foci, compared with parental cells (Figure 5(a)), suggesting that cisplatin resistance is likely established by reinforcing DNA damage repair. Moreover, the basal efficiency of HR repair was upregulated in cisplatin‐resistant cells compared with parental cells (Figure 5(b)), whereas the efficiency of NHEJ repair remained unchanged (Figure 5(c)). Therefore, elevated HR repair efficiency appears to be a major contributor to cisplatin resistance in these cells. Furthermore, either silencing E2F2 or treating TPC‐1^CR^/B‐CPAP^CR^ cells with ononin reduced HR repair efficiency (Figure 5(d)). Ononin treatment also induced DNA damage in TPC‐1^CR^ and B‐CPAP^CR^ cells (Figure 5(e)). Collectively, ononin sensitizes cells to cisplatin treatment by repressing HR repair in PTC cells. Interestingly, MDC1, a key factor of DDR, appeared to be a direct target of E2F2 based on ChIP‐Seq data (Figure 5(f)), and binding of E2F2 to the MDC1 locus was validated by qChIP analysis (Figure 5(g)). Therefore, ononin may repress HR repair through the E2F2/MDC1 axis. Consistent with its role as a transcription factor, E2F2 induced MDC1 expression in TPC‐1 and B‐CPAP cells (Figure 5(h)). The basal level of MDC1 was also increased in TPC‐1^CR^ and B‐CPAP^CR^ cells compared with parental cells (Figure 5(i)). Ononin treatment reduced MDC1 levels in TPC‐1^CR^ and B‐CPAP^CR^ cells, but this effect was largely abolished by ectopic E2F2 (Figure 5(j)). Furthermore, cisplatin‐resistant cells overexpressing MDC1 remained refractory to cisplatin even in the presence of ononin (Figure 5(k)). We also examined MDC1 and E2F2 protein levels in 12 PTC patients. Both E2F2 and MDC1 expression varied in these patients, whereas E2F2 and MDC1 are generally increased in cisplatin‐resistant patients (No.4–12) compared with patients (No 1–3) sensitive to cisplatin (Figure 5(l)). In summary, MDC1 and E2F2 contribute to cisplatin resistance in PTC. Ononin reduces MDC1 expression via E2F2, thereby suppressing HR repair and cisplatin resistance in PTC cell lines.

## 4. Discussion

The present study demonstrates that ononin effectively sensitizes PTC cells to cisplatin treatment and provides insights into the underlying molecular mechanisms. Previous studies have validated the antitumor effects of ononin in several cancers, including colorectal, breast, and lung cancer [6]. However, only a few studies have addressed the role of ononin in PTC. Our findings therefore broaden the current understanding of ononin in the field of cancer prevention and therapy. Here, we confirmed that ononin inhibits proliferation, colony formation, and EMT in PTC cells, supporting its potential role as an adjuvant therapeutic agent in PTC management.

Ononin exhibited an IC_50_ of approximately 0.30 µmol/L in PTC cell lines, indicating its potent cytotoxic effects in cancer cell lines. This result is consistent with prior research, suggesting that flavonoids possess antitumor properties by modulating signaling pathways, such as ERK/JNK/p38 pathway, MMP2/9 pathway, and Bax/Bcl‐2 pathway, which are involved in cell growth and apoptosis [16–19]. Importantly, ononin pretreatment significantly reduced the IC_50_ of cisplatin in PTC cell lines, demonstrating that ononin is able to sensitize PTC cell lines to cisplatin. In addition, ononin sensitized TPC‐1^CR^ and B‐CPAP^CR^ cells to cisplatin, which implies that ononin may serve as an adjuvant therapeutic agent in cisplatin treatment in PTC patients. Our results are in line with the previous findings that ononin displays a synergistic effect with cisplatin and enhances the effect of cisplatin [20–22].

Notably, PTC patients displayed deregulated p53‐associated genes. Upregulated p53 downstream genes indicate activated DDR pathways in PTC. Indeed, the DNA damage level was repressed in TPC‐1^CR^ and B‐CPAP^CR^ cells in our study. Furthermore, HR repair efficiency was strengthened in cisplatin‐resistant cells. The above results suggest that the activated DDR confers resistance to cisplatin. Ononin treatment induced DNA damage in cisplatin‐resistant cells by presumably inhibiting HR repair. Previous studies have identified that targeting HR or NHEJ contributes to chemotherapy effectiveness [23–27]. Therefore, ononin is a promising inhibitor of HR and can be combined with chemotherapy drugs. Ononin induces apoptosis via downregulating ERK/JNK/p38 signaling pathways [8]. JNK and ERK are known to be involved in DDR and cellular senescence regulation in cancers [28, 29]. Ononin may thereby reduce cisplatin resistance via regulating JNK and ERK signaling pathways in PTC, but more experimental validations are warranted.

In this study, we confirmed that the upregulated E2F2 was repressed by ononin. E2F2 inhibits MDM2 and thereby leads to p53 activation [15], which prevents replicative stress and DNA damage caused by various stimuli. Therefore, ononin may repress DDR and induce DNA damage by repressing p53. In addition, we firstly validated that MDC1 is a direct target of E2F2 in PTC. MDC1 is a scaffold protein and accumulates at DNA double‐strand breaks (DSBs) following γH2AX activation [30]. MDC1 helps recruit DDR factors to DSB sites and amplifies DDR signals following DNA damage [31]. Ectopic MDC1 reversed the sensitizing effect of ononin in cisplatin‐resistant PTC cell lines, which indicates that MDC1 mediates the effect of ononin. Repressing MDC1‐mediated HR repair can sensitize cancer cells to chemotherapy drugs such as 5‐FU and etoposide [32]. Therefore, ononin may restrict HR repair in cisplatin‐resistant PTC cell lines by repressing the E2F2/MDC1 axis. However, a major limitation of this study is the lack of in vivo validation of these findings.

In our experiments, ononin exerted cytotoxic and chemosensitizing effects at submicromolar concentrations, which is within a pharmacologically realistic range for a flavonoid compound. Preclinical pharmacokinetic studies in rats have reported that oral administration of ononin‐containing formulations results in detectable plasma concentrations in the low‐submicromolar range and modest oral bioavailability [33], which increases when its major active metabolite formononetin is considered. These data support the plausibility that clinically achievable ononin (and metabolite) exposures could approximate those used in our in vitro assays, particularly with optimized dosing or formulation strategies. Nevertheless, pharmacokinetic data for ononin as a single agent in humans are still lacking, and future in vivo PK/PD studies in tumor‐bearing models and clinical trials will be required to define achievable exposure levels and to validate the combination of ononin with cisplatin in PTC patients.

In conclusion, this study highlights ononin as a promising adjuvant agent that sensitizes PTC cells to cisplatin by inhibiting the E2F2/MDC1‐mediated DDR pathway. By impairing HR repair and inducing DNA damage, ononin effectively overcomes cisplatin resistance. These results provide a compelling rationale for further preclinical and clinical evaluations of ononin in combination with standard chemotherapy for PTC treatment.

## Author Contributions

Xian Deng convinced the study. Xian Deng, Xin Qian, Lian Cheng, Dehui Qiao, and Rongjia Zhang performed experiments. Xu Li performed bioinformatics analysis. Xian Deng wrote the paper.

## Funding

The authors received no specific funding for this work.

## Disclosure

All authors read and approved the final version of the manuscript.

## Ethics Statement

The authors have nothing to report.

## Conflicts of Interest

The authors declare no conflicts of interest.

## Data Availability

The data that support the findings of this study are available from the corresponding author upon reasonable request.
